# Characterizing Norovirus Transmission from Outbreak Data, United States

**DOI:** 10.3201/eid2608.191537

**Published:** 2020-08

**Authors:** Molly K. Steele, Mary E. Wikswo, Aron J. Hall, Katia Koelle, Andreas Handel, Karen Levy, Lance A. Waller, Ben A. Lopman

**Affiliations:** Emory University, Atlanta, Georgia, USA (M.K. Steele, A.J. Hall, K. Koelle, K. Levy, L.A. Waller, B.A. Lopman);; Centers for Disease Control and Prevention, Atlanta (M.E. Wikswo, A.J. Hall);; University of Georgia, Athens, Georgia, USA (A. Handel)

**Keywords:** norovirus, transmission, reproduction number, outbreaks, enteric infections, food safety, acute gastroenteritis, foodborne diseases, viruses, United States

## Abstract

Norovirus is the leading cause of acute gastroenteritis outbreaks in the United States. We estimated the basic (R_0_) and effective (R_e_) reproduction numbers for 7,094 norovirus outbreaks reported to the National Outbreak Reporting System (NORS) during 2009–2017 and used regression models to assess whether transmission varied by outbreak setting. The median R_0_ was 2.75 (interquartile range [IQR] 2.38–3.65), and median R_e_ was 1.29 (IQR 1.12–1.74). Long-term care and assisted living facilities had an R_0_ of 3.35 (95% CI 3.26–3.45), but R_0_ did not differ substantially for outbreaks in other settings, except for outbreaks in schools, colleges, and universities, which had an R_0_ of 2.92 (95% CI 2.82–3.03). Seasonally, R_0_ was lowest (3.11 [95% CI 2.97–3.25]) in summer and peaked in fall and winter. Overall, we saw little variability in transmission across different outbreaks settings in the United States.

Norovirus is the most common cause of outbreaks of acute gastroenteritis (AGE) in the United States (*1*,*2*). The Centers for Disease Control and Prevention (CDC) collects data on AGE outbreaks through the National Outbreak Reporting System (NORS). During 2009–2017, norovirus was the suspected or confirmed etiology of 47% of AGE outbreaks reported to NORS (*3*). The size and severity of outbreaks varies across different settings, times of year, and genotypes, suggesting norovirus transmissibility is variable across different outbreak settings and contexts (*4*). Generally, the transmission potential of infectious diseases is influenced by the infectiousness of the pathogen, the duration of infectiousness, and the number of susceptible contacts exposed during the infectious period (*5*).

The reproduction number is a metric for quantifying transmissibility of a pathogen. The basic reproduction number (R_0_) is the average number of secondary cases that arise from a primary case in a completely susceptible population. The effective reproduction number (R_e_) quantifies the average number of secondary cases that arise from a primary case in a population that is not completely susceptible. R_e_ varies over the course of an outbreak as the proportion of the susceptible population changes (*6*,*7*). R_0_ and R_e_ are not just metrics of the biologic properties of pathogens but also measures of the transmissibility of a pathogen within a specific population or setting (*8*,*9*). 

Several transmission modeling studies in different settings have estimated R_0_ and R_e_ of norovirus, but a large variation in these estimates occurs and R_0_ ranges from 1.1–7.2 (*10*). Much of the R_0_ variation likely is due to differences in the structures, population mixing assumptions, and data between transmission models in different settings (*10*). Generally, model estimates from community surveillance data result in an R_0_ of ≈2, but estimates from outbreak data tend to be higher and more variable. The variability of estimates from models that use outbreak data likely are driven by context; outbreaks might occur in populations that are not representative of the population as a whole and transmission likely is higher in these settings than in the community (*4*). 

We estimated R_0_ and R_e_ for thousands of norovirus outbreaks in the United States. We evaluated whether R_0_ was associated with setting, season, year, or geographic region. In addition, we assessed whether norovirus was suspected or confirmed as the cause of the outbreak.

## Methods

### Data

We obtained data on all norovirus outbreaks during 2009–2017 from NORS and CaliciNet (*11*). We defined an outbreak as >2 epidemiologically linked cases of suspected or laboratory-confirmed norovirus. NORS data consist of web-based reports of all foodborne, waterborne, and enteric disease outbreaks transmitted by contact with environmental sources, infected persons or animals, or unknown modes of transmission reported by state, local, and territorial public health agencies. This web-based reporting system collects epidemiologic information, including the dates; settings, such as long-term care facilities, child daycare facilities, hospitals, and schools; geographic location of the outbreak; the estimated total number of cases; and exposed population (*2*). For settings that report staff and guest case numbers, we included these data in the estimated total number of cases and exposed population. CaliciNet data consists of sequence-derived genotypes and epidemiologic data from norovirus outbreaks submitted from local, state, and federal public health laboratories. We obtained CaliciNet genotypes that were linked to outbreak data we acquired from NORS.

For all outbreaks reported to NORS, data are collected on the total estimated primary cases, including all laboratory-confirmed and suspected primary cases. These data exclude cases associated with secondary illnesses, such as person-to-person norovirus transmission in households after a restaurant-based outbreak. However, data for calculating attack rates, specifically the number of exposed persons and the subset of the exposed persons who became ill, are only collected for outbreaks with person-to-person, environmental, or unknown transmission modes. In addition, data collected from outbreaks might not be documented consistently across a report. For example, outbreaks for which setting-specific information on the total number of guests and staff that are reported to be ill, referred to as total ill, might not match the reported total estimated primary cases. During 2009–2017, a total of 17,822 suspected and confirmed norovirus outbreaks were reported to NORS. We excluded 10,728 outbreaks based on the following criteria, which we imposed hierarchically: transmission was not person-to-person (n = 3,866); the outbreak exposure occurred in multiple states (n = 8); the outbreak occurred in Puerto Rico (n = 3), which we excluded because of small sample size; the size of total exposed population or major setting were not reported (n = 5,573); the total estimated primary cases and the total ill among the exposed population were not equal (n = 1,231); or the total estimated primary cases or the total ill among the exposed population were reported to be greater than the total exposed population size (n = 47) (Appendix Figure 1). In all, 7,094 norovirus outbreaks met our inclusion criteria in subsequent analyses (Appendix Table 1). We did not use imputation techniques to infer values for missing data because no good proxy variables inferred missing data for major settings and exposed population size.

### Estimating R_0_ and R_e_

We used the final size method to calculate R_0_, R_e_, and associated SEs (*12*; Appendix). The final size method calculates R_0_ and R_e_ based on 3 variables: the total population size of the outbreak (*N*), the total number of cases in the outbreak (*C*), and the number of susceptible persons at the start of the outbreak (*S*). In our calculations, *C* was informed by NORS outbreak data for the estimated total number ill and *N* by the exposed population. NORS data does not include nor can it inform the number of susceptible persons at the start of an outbreak. Therefore, to estimate *S*, we used norovirus challenge study data on the percent of persons that become infected and develop AGE after challenge with virus. Across all published studies, the weighted average of participants in whom gastroenteritis developed after challenge is 47% (range 27%–80%; Appendix Table 1) (*13*–*19*). We assumed *S* is the number of persons susceptible to disease, as opposed to infection. To calculate *S*, we multiplied 47% by *N* and rounded to the nearest integer. For 890 outbreaks, the total number of cases, *C*, was greater than our estimated *S*; for these outbreaks we set *S* equal to *C*, corresponding to a 100% attack rate. We also calculated *S* assuming 27% and 80% of *N* were susceptible to assess the sensitivity of our model results to this parameter.

### Regression Analysis

After estimating R_0_, R_e_, and associated SEs for each norovirus outbreak, we fit a linear regression model to the log-transformed estimated reproduction numbers to assess whether outbreak setting, census region, season, year, suspected or confirmed norovirus, or genotype were associated with transmissibility. All variables were categorical, where the reference was assigned as the group with the most outbreaks reported, except for the suspected or confirmed variable, for which we set the referent to outbreaks with confirmed norovirus etiology. We used weighted least squares combined with estimated standard errors to produce robust estimates accounting for heteroscedasticity and non–normally distributed model residuals by using the estimatr package in R version 3.4.2 (*20*,*21*). We included the following variables in our models: outbreak setting; census region; meteorological season, defined as spring (March 1–May 31), summer (June 1–August 31), fall (September 1–November 30), or winter (December 1–February 28); year, defined as July–June; whether norovirus was suspected or confirmed; and norovirus genotype, categorized as GI, GII.4, or GII.non4. 

For outbreaks for which we calculated R_0_ and R_e_, we had norovirus genotype data for only 22% (1,571). In a preliminary analysis, we fit a univariate linear regression model to estimate R_0_ by norovirus genotype alone and by norovirus genotype and year and found no evidence for variation (Appendix). Given these results and the small sample size, we did not include norovirus genotype in our models and performed model selection on the remaining variables. To determine which variables to include, we used a forward selection process and selected the model with the lowest Akaike information criterion and Bayes information criterion values.

### Sensitivity Analysis

We tested the sensitivity of our regression model results to different modeling approaches and different assumptions of the percent susceptible at the start of an outbreak. We also fit a logistic regression model of binary transmission and a negative binomial regression of the final outbreak size by using the log-transformed exposed population size as a measure of the attack rate of an outbreak. Thus, we could make comparisons between the models to see if the results from modeling continuous transmission were consistent with the results of modeling binary transmission and attack rates. In addition, we ran all the regression models again using the assumption that 27% and 80% susceptible at the start of an outbreak, which corresponds to the minimum and maximum percent susceptible to AGE from published challenge studies (Appendix Table 2).

## Results

Of the 7,094 norovirus outbreaks included in our final dataset, 75% (5,335) occurred in long-term care and assisted living facilities and 57% (4,016) occurred in winter. The median outbreak size was 28 cases (interquartile range [IQR] 16–47) and the median attack rate was 22% (IQR 11%–36%) (Table 1). The median R_0_ was 2.75 (IQR 2.38–3.65) and the median R_e_ was 1.29 (IQR 1.12–1.74).

**Table 1:** Norovirus outbreaks with exposed population size reported to the National Outbreak Reporting System, United States, 2009–2017*

Characteristics	No. (%)	Median attack rate, % (IQR)	Median final size, % (IQR)	Median R_0_ (IQR)
All outbreaks	7,094 (100)	22 (11–36)	28 (16–47)	2.75 (2.38–3.65)
Major setting				
Child day care	272 (4)	21 (13–36)	18 (11–29)	2.67 (2.39–3.60)
Hospital or healthcare facility	271 (4)	22 (11–38)	19 (11–34)	2.70 (2.33–3.59)
Long-term care or assisted living facility	5,335 (75)	23 (13–36)	30 (17–47)	2.81 (2.42–3.76)
Other	350 (5)	20 (10–36)	24 (15–40)	2.66 (2.35–3.60)
Private home or residence	42 (1)	66 (50–91)	9 (6–16)	3.80 (2.26–4.92)
Restaurant	77 (1)	50 (27–64)	10 (6–16)	3.12 (2.53–4.31)
School, college, or university	747 (11)	12 (6–24)	42 (19–80)	2.41 (2.24–2.92)
Season				
Winter	4,016 (57)	22 (12–36)	30 (17–51)	2.80 (2.40–3.77)
Fall	808 (11)	21 (11–37)	26 (15–47)	2.72 (2.36–3.63)
Spring	1,964 (28)	20 (11–35)	27 (15–44)	2.69 (2.37–3.57)
Summer	306 (4)	17 (9–33)	19 (11–32)	2.57 (2.29–3.33)
Outbreak status				
Confirmed	3,114 (44)	26 (15–40)	35 (20–55)	2.99 (2.51–4.22)
Suspected	3,980 (56)	18 (9–31)	24 (14–40)	2.59 (2.32–3.27)
Census region				
Northeast	1,898 (27)	17 (9–29)	31 (17–53)	2.58 (2.30–3.23)
Midwest	2,205 (31)	25 (13–39)	26 (15–44)	2.87 (2.44–3.98)
South	2,224 (31)	23 (12–38)	29 (17–47)	2.81 (2.39–3.93)
West	767 (11)	21 (13–34)	28 (16–44)	2.75 (2.42–3.57)
Year				
2009 Jan–Jun†	243 (3)	28 (15–42)	35 (20–55)	3.09 (2.50-4.56)
2009 Jul–2010 Jun	275 (4)	29 (15–45)	35 (19–57)	3.17 (2.51–4.77)
2010 Jul–2011 Jun	592 (8)	29 (16–44)	32 (19–54)	3.12 (2.54–4.58)
2011 Jul–2012 Jun	679 (10)	27 (15–40)	35 (19–59)	3.01 (2.52–4.29)
2012 Jul–2013 Jun	967 (14)	21 (12–36)	28 (16–46)	2.73 (2.38–3.61)
2013 Jul–2014 Jun	913 (13)	20 (11–33)	29 (18–51)	2.68 (2.38–3.45)
2014 Jul–2015 Jun	941 (13)	21 (11–35)	28 (16–46)	2.74 (2.3–3.61)
2015 Jul–2016 Jun	1,007 (14)	17 (9–32)	25 (14–42)	2.57 (2.31–3.29)
2016 Jul–2017 Jun	1,070 (15)	19 (10–31)	26 (14–42)	2.63 (2.33–3.24)
2017 Jul–Dec†	407 (6)	20 (10–34)	22 (14–38)	2.66 (2.33–3.55)

*IQR, interquartile range. †Partial norovirus years included in this analysis. The National Outbreak Reporting System was established in January 2009, and the first year of this analysis is 2009 January–June. At the time of analysis, we received data through December 2017.

### Model Selection and Regression Analysis

The final selected model included the following variables: major setting, census region, season, year, and whether norovirus was suspected or confirmed (Akaike information criterion = 5,803; Bayes information criterion = 5,968) (Appendix Table 3). For long-term care and assisted living facilities, R_0_ was 3.35 (95% CI 3.26–3.45). R_0_ for outbreaks in all other settings did not differ substantially, except for outbreaks in schools, colleges, and universities, in which R_0_ was slightly reduced, 2.92 (95% CI 2.82–3.03) (Table 2; Appendix Figure 2). We found that R_0_ differed substantially by outbreak status; suspected norovirus outbreaks had a lower R_0_, 3.02 (95% CI 2.94–3.10), than that for confirmed outbreaks (R_0_ = 3.35 [95% CI 3.26–3.45]).

**Table 2:** Estimated log-linear change in R_0_ from the intercept for linear regression of log transformed R_0_ for norovirus outbreaks reported to the National Outbreak Reporting System, United States, 2009–2017

Category	Estimated log-linear change in R_0 _(95% CI)
Intercept	3.35 (3.26–3.45)
Major setting	
Long-term care or assisted living facility	Referent
Child day care	0.99 (0.95–1.03)
Hospital or healthcare facility	0.93 (0.90–0.97)
Other	0.97 (0.93–1.01)
Private home or residence	0.99 (0.82–1.19)
Restaurant	1.01 (0.91–1.11)
School, college, or university	0.87 (0.85–0.89)
Season	
Winter	Referent
Fall	1.00 (0.98–1.03)
Spring	0.98 (0.96–1.00)
Summer	0.93 (0.89–0.96)
Outbreak status	
Confirmed	Referent
Suspected	0.90 (0.88–0.92)
Census region	
South	Referent
Northeast	0.89 (0.87–0.91)
Midwest	1.00 (0.97–1.02)
West	0.98 (0.95–1.01)
Year	
2009 Jan–Jun	1.16 (1.10–1.23)
2009 Jul–2010 Jun	1.17 (1.11–1.23)
2010 Jul–2011 Jun	1.16 (1.12–1.21)
2011 Jul–2012 Jun	1.12 (1.08–1.16)
2012 Jul–2013 Jun	1.04 (1.01–1.07)
2013 Jul–2014 Jun	1.02 (0.99–1.06)
2014 Jul–2015 Jun	1.05 (1.02–1.08)
2015 Jul–2016 Jun	1.02 (0.99–1.05)
2016 Jul–2017 Jun	Referent
2017 Jul–Dec	1.04 (1.00–1.09)

Estimated R_0_ varied only slightly by census region and was lowest in the northeast (R_0_ = 3.00 [95% CI 2.92–3.08]). Season and year also contributed to changes in the R_0_. Estimated R_0_ was highest in winter (3.35 [95% CI 3.26–3.45]) and fall (3.37 [95% CI 3.24–3.50]) and lowest during the summer months (3.11 [95% CI 2.97–3.25]). Outbreaks reported during January 2009–June 2012 all had higher estimated R_0_ (range for individual seasonal years 3.77–3.93) than the reference period, July 2016–June 2017 (Table 2; Appendix Figure 2). Our findings were generally robust to assumptions about the proportion susceptible at the start of the outbreak and whether we modeled the outcome of R_0_, R_e_, or final outbreak size (Appendix Tables 4–6, Figure 3).

## Discussion

By using a large national outbreak dataset, we investigated transmission patterns of norovirus outbreaks. Our analysis led to several key findings. First, reported norovirus outbreaks in the United States have modest R_0_ (2.75 [IQR 2.38–3.65]) and R_e_ (1.29 [IQR 1.12–1.74]) values. Second, we found that R_0_ and R_e_ did not vary across most settings, except for outbreaks in schools, colleges, and universities, which had lower estimated transmission values. Third, we found higher transmission in laboratory-confirmed outbreaks relative to suspected outbreaks and higher transmission for outbreaks occurring in the winter months relative to summer months.

Our finding that norovirus outbreaks in the United States have modest transmission values is somewhat surprising. In a recent review of norovirus modeling studies, Gaythorpe et al. (*10*) found R_0_ estimates for norovirus were 1.1–7.2. Of note, R_0_ and R_e_ estimates from transmission modeling studies that analyzed data from norovirus outbreaks were high, but variability between studies was high; R_e_ estimates were ≈1–14 (*22*–*24*). Our estimates are within the reproduction numbers estimated by using transmission models of norovirus based on outbreak data (*22*,*25*). However, our estimates are higher than those from several studies that estimated reproduction numbers by using population-level transmission models (*26*–*29*), suggesting that transmission of norovirus in outbreak settings is higher than sporadic transmission in the community.

From our main analysis, we found that outbreaks in schools, colleges, and universities had lower estimated transmission, but transmission varied little across all other settings. Relative to outbreaks in long-term care and assisted living facilities, outbreaks that occurred in private homes or residences and restaurants had higher final sizes, and schools, colleges, and universities had lower estimated attack rates. Our finding that outbreaks in the winter had higher estimated transmissibility than outbreaks that occurred in summer is likely a factor of the strong wintertime seasonality of noroviruses in the United States (*30*,*31*). Consistent with this finding are the observations that norovirus case and outbreak reports are inversely correlated with temperature (*30*,*31*) and that survival of norovirus surrogate viruses, such as murine norovirus and feline calicivirus, declines with increasing temperatures (*32*,*33*).

Several differences we found might be driven by surveillance biases rather than differences in norovirus transmission. Suspected norovirus outbreaks without a laboratory-confirmed outbreak etiology had lower transmission than laboratory-confirmed norovirus outbreaks, perhaps because suspected outbreaks are not investigated as well as confirmed outbreaks and have lower rates of case ascertainment. Outbreaks reported in the south had higher estimated R_0_ and R_e_ relative to outbreaks in the northeast, which might be related to differences in the quality of reporting between these regions. For example, if surveillance in certain regions only captured larger, more easily detectable outbreaks with higher attack rates, this could bias our estimates of transmissibility upwards. Tremendous variability exists in outbreak reporting between states, ≈100-fold difference between the highest and lowest reporting states, which likely affects the observed outbreak characteristics we included (*34*). Similarly, NORS has been collecting outbreak reports since January 2009, but in August 2012 CDC began a concerted effort to improve norovirus outbreak reporting to NORS and CaliciNet with the introduction of NoroSTAT (*35*,*36*). Thus, our finding that norovirus outbreaks reported before August 2012 were larger and had higher estimated R_0_ and R_e_ values might be related to CDC’s efforts to capture outbreaks that previously would not have been reported, such as smaller outbreaks. Further, because the transmission mode can be difficult to identify for norovirus outbreaks, our analysis might have included outbreaks for which the mode of transmission was misclassified as person-to-person. Larger outbreaks with higher transmission are more likely to be reported, and our results might not reflect transmission in smaller outbreaks. In addition, the exposed population size is difficult to quantify and is not consistently reported to NORS. Thus, the differences we found in estimated attack rates across different settings could be due to true variability in the exposed population size across settings or variability in the reliable reporting of the exposed population size. However, our analysis restricted to outbreaks in long-term care and assisted living facilities found the same trends among the variables for outbreak status, census region, season, and year as our analysis of all outbreaks, which suggests the results are robust.

Our study has several additional limitations. First, our process of data selection might have introduced bias into our analyses. We excluded outbreaks that occurred in multiple states, which are likely to have higher transmissibility given the larger geographic range involved; however, only 8 multistate outbreaks occurred during the study period, thus the bias is likely negligible. A substantial proportion of the dataset, 5,573 (31%) outbreaks, had to be excluded because the exposed population size was not reported. Excluding these outbreaks could introduce bias if the exposed population size is more likely to be reported for outbreaks with smaller, or larger, exposed population sizes. We only included outbreaks with person-to-person transmission; thus, our estimates of transmissibility are not generalizable to norovirus outbreaks where transmission occurs via other modes, such as foodborne, waterborne, or environmental transmission.

A second set of limitations relates to the final size method. This method assumes a susceptible-infected-recovered type infection in a homogenously mixing population (*12*), but this simplification likely does not reflect true mixing patterns. In addition, we might observe different mixing patterns in each of the different outbreak settings, such as older persons in long-term care facilities versus young children in childcare. The final size method also underestimates reproduction numbers for outbreaks with high attack rates. For example, in private homes, attack rates are high, but exposed population sizes are small. If everyone in the household is infected, then no additional infections can occur in the home. Thus, the final size method cannot capture any additional transmission that could have happened if the exposed population size had been larger, such as a higher number of persons in the household. Becker termed this limitation the “wasted infection potential” (*37*). Further, the final size method does not account for the effect of control measures. For some of the outbreaks represented in our dataset, control measures were most likely implemented, such as isolating ill persons and cleaning contamination. Such interventions likely would reduce the number ill, and the estimated R_0_ would be lower than the R_0_ in the absence of control measures.

In addition, the final size method assumes that the proportion of susceptible persons is known at the start of an outbreak; however, the level of susceptibility to norovirus in not well known. Certain host genetic factors are associated with the ability of norovirus to establish an infection within a human host (*38*–*41*), leading to variable susceptibility to norovirus infection (*42*–*44*). Secretor-negative persons have nonfunctional fucosyltransferase-2 genes, causing infection failure for norovirus genogroups I and II type 4 (*38*,*40*,*41*,*45*,*46*). Our estimates of R_0_ and R_e_ assume that 47% of the population in our dataset is susceptible at the start of all outbreaks. However, the proportion susceptible varies among outbreaks and potentially over time and age as the distribution of circulating norovirus genotypes change. Further, our regression model estimates were sensitive to our assumption of the percent susceptible at the start of an outbreak. When we assumed 47% and 80% of the population was susceptible, the estimated transmissibility of norovirus in private homes or residences and restaurants was higher than transmissibility in long-term care and assisted living facilities. However, when we assumed 27% of the population was susceptible at the start of an outbreak, the association between private homes or residences and restaurants reversed. These settings then had lower estimated transmission relative to outbreaks in long-term care and assisted living facilities because the population size that can be infected is much lower, thus reducing the estimates of R_0_ and R_e_. For example, if a household had 15 persons, the maximum possible R_0_ assuming 27% susceptibility is 4, which is lower than the average predicted R_0_ for outbreaks in the reference group. Therefore, the results for private homes or residences and restaurants, where exposed population sizes are lower, should be interpreted with caution because transmission values in these settings might be underestimated. We also assumed that only symptomatic persons contribute to transmission in our calculation; persons with asymptomatic norovirus infections can contribute to transmission, but they likely are not as infectious as persons with symptomatic infections (*22*,*47*).

Finally, our main analysis does not account for norovirus genotype. Because of the limited data available on outbreak genotype we were not able to fully assess whether certain genotypes were more transmissible. As more genotyping data become available, future studies should investigate transmissibility.

We estimated reproduction numbers by using the final size method for >7,000 outbreaks from a national outbreak reporting system, then used these estimates to examine factors associated with norovirus transmission. Our analyses suggest that norovirus transmission rates are modest. Such modest rates of R_e_ suggest there are opportunities for effective control measures to curtail transmission of norovirus. However, challenges remain. Transmission by asymptomatic persons, which we did not account for in this analysis and generally goes undetected in surveillance, can limit the effectiveness of traditional control methods focused on ill persons, even for pathogens with modest transmission (*48*).

Overall, we found limited variation in R_0_ and R_e_ for reported norovirus outbreaks in the United States, particularly across different settings. Our findings highlight the need for better data on the total exposed population sizes in outbreaks, which heavily influence estimates of attack rates, R_0_, and R_e_, to further refine estimates of these outbreak factors.

Appendix Additional information on characterizing norovirus transmission from outbreak data, United States.
