# Supplementary material for: Characterizing Norovirus Transmission from Outbreak Data, United States
Source: Emerg Infect Dis. 2020 Aug;26(8):1818–25. doi: 10.3201/eid2608.191537 (PMC7392428; doi:10.3201/eid2608.191537)
Supplement: Appendix — Additional information on characterizing norovirus transmission from outbreak data, United States. [file 19-1537-Techapp-s1.pdf]

Article DOI: https://doi.org/10.3201/eid2608.191537

Characterizing Norovirus Transmission from Outbreak Data, United States

Appendix

Estimating Basic and Effective Reproduction Numbers

We calculated basic reproduction (R_0), effective reproduction (R_e), and the associated standard errors (SE) for each outbreak. We used equations proposed by Becker (*1*) that use the final epidemic size

$$R_0 = \frac{N-1}{C} \sum_{i=S-C+1}^{S} \frac{1}{i}$$

$$SE(R_0) = \frac{N-1}{C} \sqrt{\sum_{i=S-C+1}^{S} \frac{1}{i^2} + \frac{CR_0^2}{(N-1)^2}}$$

where N is the total population size, C is the total number of cases in the outbreak, and S is the number of susceptible persons at the start of the outbreak. R_e is calculated by replacing N with S in the first equation above. SE(R_e) is calculated by replacing N with S and R_0 with R_e in the second equation shown above. The final size method assumes a susceptible-infected-recovered type infection with a closed, homogenously mixing population (*1*).

Genogroup and genotype analysis

Among the outbreaks for which we calculated R_0 and R_e only 1,571 outbreaks (22%) had data on norovirus genotype. We fit a linear regression model to the log transformed estimate of R_0 to assess whether the following genogroup and genotypes were associated transmissibility: genogroup I (GI), genogroup II genotype 4 (GII.4) and genogroup II non-genotype 4 (GII.non4). We found that R_0 varied little for outbreaks of GI (R_0 = 3.50 [95% CI 3.32, 3.68]), GII.4 (R_0 = 3.46 [95% CI 3.38, 3.55]) and GII.non4 (R_0 = 3.26 [95% CI 3.11, 3.41]).

In addition to the univariate regression analysis of genogroup and genotype, we also fit a linear regression model to the log transformed estimate of R_0 with predictor variables for

genotype and year to assess whether GII.4 noroviruses had different effects on transmissibility at the time when new strains emerged (i.e., GII.4 New Orleans in the 2009/2010 season and GII.4 Sydney in the 2012/2013 season). There was not sufficient evidence to suggest that R_0 differed for outbreaks of GII.4 during the norovirus seasons when GII.4 New Orleans (R_0 = 4.00 [95% CI 3.53, 4.54]) or GII.4 Sydney (R_0 = 3.38 [95% CI 3.24, 3.53]) relative to GII.4 outbreaks reported in seasons when no new strains emerged (R_0 = 3.38 [95% CI 3.19, 3.58]). (Appendix Table 4)

Alternative Models

We assessed two alternative approaches for modeling norovirus transmission: a logistic regression to model a binary transmission outcome (i.e., high versus low transmission) and a negative binomial regression to model the final size of outbreaks, adjusting for exposed population size (i.e., modeling attack rates). For our logistic regression, we used the first and third tertiles of estimated values of R_0 and R_e, assuming the percent susceptible was 47%, to determine the cutoffs for our outcome of interest: low versus high transmission. We excluded outbreaks with transmission values within the second tertile and focus our logistic regression comparison between the lowest and highest tertiles of transmission. The third tertile of R_0 and R_e values were 3.23 and 1.52, respectively.

The trends of transmissibility across our variables of interest (outbreak setting, census region, season, year, whether norovirus was suspected or confirmed and norovirus genotype) from our main regression analysis of a continuous transmission outcome were consistent across the logistic regressions of high R_0 (R_0>3.23) and R_e (R_e>1.52) and linear regression of R_e values. (Appendix Table 5) The trends of transmissibility were consistent for most of our variables of interest in the negative binomial model of final outbreak sizes; however, private homes or residences and restaurants had a much more pronounced effect on the attack rate of outbreaks, relative to long-term care and assisted living facilities (RR = 2.35 (95% CI 1.85, 3.01) and RR = 1.67 (95% CI 1.40, 2.01), respectively). As the exposed population size is difficult to quantify, and thus may not be reported reliably, we analyzed the subset of outbreaks that occurred within long-term care and assisted living facilities with our regression models. The patterns found among the variables for outbreak status, census region, season and year were consistent with what was found analyzing the full dataset. (Appendix Table 6)

Percent Susceptible

We tested the sensitivity of our results to the assumption of the percent susceptible start of an outbreak by running all the regression models assuming the percent susceptible at the start was 27% and 80%, which represent the minimum and maximum estimates of the percent susceptible to AGE from published challenge studies, respectively (Appendix Table 2). By adjusting our assumption of the percent susceptible at the start of the outbreak to 27% and 80%, the median R_0 was 6.04 (IQR 4.53, 9.38) and 1.43 (IQR 1.33, 1.61), respectively, while the median R_e was 1.82 (IQR 1.24, 3.83) and 1.14 (IQR 1.07, 1.29), respectively. Assuming the proportion susceptible was 27% we found that outbreaks in long-term care and assisted living facilities were more likely to have R_0 >8.05 and R_e>3.24 relative to all other settings. (Appendix Table 5, Appendix Table 6, Appendix Figure 2) When the percent susceptible was 80% we found that outbreaks in long-term care and assisted living facilities had increased odds of having R_0 >1.54 and R_e>1.23 compared to hospitals/other healthcare facilities, schools/colleges/universities, and other settings. Outbreaks in private homes or residences and restaurants had higher odds of having R_0 >1.54 and R_e>1.23 relative to long-term care and assisted living facilities, however the confidence intervals are wide due to small sample sizes. (Appendix Table7, Appendix Table 8, Appendix Figure 2) Trends in the variables for census region, season, year, and whether norovirus was suspected or confirmed for the models assuming 27% and 80% susceptibility were consistent with the models assuming 47% susceptibility. (Table 2, Appendix Table 7, Appendix Table 8)

Appendix Table 1. Descriptive characteristics of norovirus outbreaks that met our inclusion criteria and those outbreaks that were excluded from this analysis

Characteristics	No. (%)	Median attack rate (IQR)*	Median final size (IQR)
All Outbreaks	10,728 (60)	22 (10–44)	20 (10–39)
Major setting			
Child day care	168 (2)	21 (16–36)	16 (10–26)
Hospital/healthcare facility	316 (3)	24 (9–54)	16 (8–28)
Long-term care/assisted living facility	3,596 (34)	21 (10–42)	25 (14–43)
Other	493 (5)	24 (11–42)	19 (9–40)
Private home/residence	81 (1)	61 (40–95)	8 (5–13)
Restaurant	259 (2)	41 (17–68)	8 (4–13)
School/college/university	562 (5)	12 (6–26)	30 (13–69)
Missing	5,253 (49)	30 (14–43)	18 (8–36)
Season			
Winter	5,597 (49)	22 (10–4)	23 (11–44)
Fall	1,390 (13)	20 (10–40)	18 (8–35)
Spring	3,137 (30)	21 (10–43)	19 (9–36)
Summer	868 (8)	23 (10–41)	14 (7–26)
Outbreak Status			
Confirmed	4,875 (55)	22 (11–42)	23 (11–42)
Suspected	5,853 (45)	21 (9–44)	18 (9–36)
Census region			
Northeast	2,667 (25)	11 (6–24)	24 (12–47)

Characteristics	No. (%)	Median attack rate (IQR)*	Median final size (IQR)
Midwest	3,964 (37)	33 (17–62)	19 (9–36)
South	1,863 (17)	21 (10–40)	22 (10–45)
West	2,207 (21)	25 (13–41)	17 (8–32)
Multistate	16 (0.1)	30 (10–39)	14 (8–54)
Puerto Rico	11 (0.1)	12 (7–24)	18 (8–30)
Year			
2009 Jan–Jun†	630 (6)	32 (20–55)	25 (13–46)
2009 Jul–2010 Jun	914 (9)	22 (10–41)	22 (10–39)
2010 Jul–2011 Jun	1,079 (10)	29 (14–46)	25 (13–48)
2011 Jul–2012 Jun	1,107 (10)	15 (6–35)	22 (10–47)
2012 Jul–2013 Jun	1,405 (13)	23 (9–51)	22 (10–40)
2013 Jul–2014 Jun	1,240 (12)	21 (9–43)	20 (9–39)
2014 Jul–2015 Jun	1,224 (11)	25 (13–46)	20 (9–38)
2015 Jul–Jun 2016	1,279 (12)	17 (9–38)	17 (8–32)
2016 Jul–2017 Jun	1,397 (13)	21 (10–37)	18 (9–34)
2017 Jul–Dec†	456 (40)	21 (10–41)	16 (8–31)
Genogroup and genotype			
GII.4	1,550 (14)	27 (13–48)	23 (11–38)
GII.non4	774 (7)	23 (11–44)	19 (9–34)
GI	436 (4)	26 (15–53)	21 (10–40)
Missing	7,968 (74)	20 (9–41)	20 (10–10)

***The medians and IQRs for attack rates were calculated for the subset of outbreaks where both the exposed population size and total estimated primary cases were reported. Among the 10,728 outbreaks excluded from our primary analysis, 8,903 outbreaks were missing data for the exposed population size.

†Two partial norovirus years included in this analysis. NORS was established in January 2009, thus the first year of this analysis is January–June 2009. At the time of analysis, we received data through December 2017, thus the final year of this analysis is July–Dec 2017.

Appendix Table 2. Data from published norovirus challenge studies on the number of participants challenged with norovirus and the number of challenged participants who subsequently developed acute gastroenteritis*

			Acute gastroenteritis	
Study (reference)	Secretor-negative screening	No. challenged	No.	Proportion
Dolin 1972 (*2*)	Secretors not screened	12	9	0.75
Wyatt 1974 (*3*)	Secretors not screened	23	16	0.70
Parrino 1977 (*4*)	Secretors not screened	12	6	0.50
Treanor 1988 (*5*)	Secretors not screened	10	8	0.80
Johnson 1990 (*6*)	Secretors not screened	42	25	0.60
Graham 1994 (*7*)	Secretors not screened	50	34	0.68
Lindesmith 2003 (*8*)	Secretor-negatives included	77	21	0.27
Lindesmith 2005 (*9*)	Secretor-negatives included	15	7	0.47
Atmar 2008 (*10*)	Secretor-negatives excluded	21	11	0.52
Leon 2011 (*11*)	Secretor-negatives excluded	15	5	0.33
Atmar 2011 (*12*)	Secretor-negatives excluded	41	29	0.71
Seitz 2011 (*13*)	Secretor-negatives excluded	13	10	0.77
Frenck 2012 (*14*)	Secretor-negatives included	40	12	0.30
Bernstein 2015 (*15*)	Secretor-negatives excluded	98	29	0.30
Overall		469	222	0.47†

*We assume that the average proportion that develop AGE across all studies, weighted by total number of participants in each study, is the proportion that are susceptible to norovirus in our calculations of R_0 and R_e.

†Average proportion susceptible weighted by number of participants.

Appendix Table 3. Estimated log linear change in R_0 (95% CI) from the estimated R_0 for the intercept for each model in a forward selection process for a linear regression model of log transformed R_0 values*

Model variable	Model 1	Model 2	Model 3	Model 4	Model 5
Intercept	3.22 (3.19–3.25)	3.44 (3.39-3.49)	3.57 (3.50–3.64)	3.61 (3.53–3.68)	3.35 (3.26–3.45)
Child day care	0.95 (0.91–0.99)	0.99 (0.95–1.03)	0.98 (0.94–1.02)	0.98 (0.94–1.02)	0.99 (0.95–1.03)
Hospital or healthcare facility	0.94 (0.90–0.98)	0.93 (0.89–0.97)	0.94 (0.90–0.98)	0.94 (0.90–0.98)	0.93 (0.90–0.97)
Other	0.96 (0.92–1.00)	0.96 (0.93–1.00)	0.95 (0.91–0.99)	0.97 (0.93–1.01)	0.97 (0.93–1.01)
Private home or residence	1.00 (0.84–1.19)	0.99 (0.83–1.18)	0.98 (0.81–1.17)	0.99 (0.82–1.19)	0.99 (0.82–1.19)
Restaurant	1.02 (0.93–1.12)	1.01 (0.92–1.11)	1.00 (0.91–1.09)	1.00 (0.91–1.10)	1.01 (0.91–1.11)
School, college, or university	0.84 (0.82–0.87)	0.88 (0.86–0.90)	0.86 (0.84–0.88)	0.86 (0.84–0.88)	0.87 (0.85–0.89)
Suspected outbreak	–	0.88 (0.87–0.90)	0.88 (0.87–0.90)	0.89 (0.87–0.90)	0.90 (0.88–0.92)
Region 1	–	–	0.89 (0.87–0.91)	0.89 (0.87–0.91)	0.89 (0.87–0.91)
Region 2	–	–	0.99 (0.97–1.02)	0.99 (0.97–1.02)	1.00 (0.97–1.02)
Region 4	–	–	0.96 (0.93–0.99)	0.96 (0.93–0.99)	0.98 (0.95–1.01)
Fall	–	–	–	1.00 (0.97–1.03)	1.00 (0.98–1.03)
Spring	–	–	–	0.97 (0.95–0.99)	0.98 (0.96–1.00)
Summer	–	–	–	0.92 (0.88–0.95)	0.93 (0.89–0.96)

Model variable	Model 1	Model 2	Model 3	Model 4	Model 5
Jan 2009–Jun 2009	–	–	–	–	1.16 (1.10–1.23)
Jul 2009–Jun 2010	–	–	–	–	1.17 (1.11–1.23)
Jul 2010–Jun 2011	–	–	–	–	1.16 (1.12–1.21)
Jul 2011–Jun 2012	–	–	–	–	1.12 (1.08–1.16)
Jul 2012–Jun 2013	–	–	–	–	1.04 (1.01–1.07)
Jul 2013–Jun 2014	–	–	–	–	1.02 (0.99–1.06)
Jul 2014–Jun 2015	–	–	–	–	1.05 (1.02–1.08)
Jul 2015–Jun 2016	–	–	–	–	1.02 (0.99–1.05)
Jul 2017–Dec 2017	–	–	–	–	1.04 (1.00–1.09)
Fitting Metrics					
Akaike information criterion	6,237	6,049	5,935	5,920	5,803
Bayes information criterion	6,291	6,111	6,017	6,023	5,968

*The Akaike information criterion, Bayes information criterion, and adjusted R^2 are presented for each model.

Appendix Table 4. Estimated log-linear change in R_0 from the intercept for linear regression of log transformed R_0 by genotype and year

Characteristics	Estimated log-linear change in R_0 (95% CI)
Intercept	3.38 (3.19–3.58)
Genogroup or genotype	
GII.4	Referent
GI	1.01 (0.95–1.07)
GII.non4	0.94 (0.89–0.99)
Year	
Jan 2009–Jun 2009	0.98 (0.76–1.25)
Jul 2009–Jun 2010	1.18 (1.03–1.35)
Jul 2010–Jun 2011	1.11 (1.01–1.21)
Jul 2011_Jun 2012	1.00 (0.93–1.08)
Jul 2012–Jun 2013	1.00 (0.93–1.07)
Jul 2013–Jun 2014	1.02 (0.95–1.10)
Jul 2014–Jun 2015	1.02 (0.95–1.10)
Jul 2015–Jun 2016	0.98 (0.91–1.06)
Jul 2016–Jun 2017	Referent
Jul 2017–Dec 2017	1.14 (1.01–1.28)

Appendix Table 5. Risk ratios of attack rates, estimated log-linear change in R_0 and R_e relative to the intercept from linear regression of the log transformed reproduction numbers and odds ratios of an outbreak with high transmission from logistic regression*

Characteristics	RR of attack rates (95% CI)	OR of R_0>3.23 (95% CI)	Estimated log-linear change in R_e (95% CI)	OR of R_e>1.52 (95% CI)†
Intercept	0.27 (0.26–0.29)	1.75 (1.41–2.18)	1.63 (1.58–1.68)	1.72 (1.39–2.13)
Major setting				
Long-term care, assisted living facility	Referent	Referent	Referent	Referent
Child day care	1.08 (0.98–1.19)	1.03 (0.74–1.42)	1.00 (0.95–1.05)	1.11 (0.80–1.53)
Hospital or healthcare facility	0.94 (0.86–1.03)	0.62 (0.45–0.85)	0.94 (0.89–0.98)	0.65 (0.48–0.89)
Other	0.98 (0.90–1.06)	0.73 (0.55–0.96)	0.98 (0.93–1.02)	0.74 (0.56–0.98)
Private home or residence	2.35 (1.85–3.01)	1.80 (0.88–3.87)	1.31 (1.14–1.51)	8.47 (3.22–29.27)
Restaurant	1.67 (1.40–2.01)	1.41 (0.78–2.62)	1.10 (0.99–1.22)	1.96 (1.09–3.67)
School, college, or university	0.67 (0.63–0.71)	0.29 (0.23–0.36)	0.86 (0.83–0.88)	0.30 (0.24–0.38)
Season				
Winter	Referent	Referent	Referent	Referent
Fall	0.99 (0.94–1.05)	1.00 (0.81–1.23)	1.01 (0.97–1.04)	0.97 (0.79–1.19)
Spring	0.97 (0.93–1.01)	0.90 (0.78–1.04)	0.98 (0.95–1.00)	0.89 (0.77–1.04)
Summer	0.86 (0.79–0.95)	0.65 (0.47–0.88)	0.92 (0.88–0.96)	0.62 (0.45–0.85)
Outbreak Status				
Confirmed outbreak	Referent	Referent	Referent	Referent
Suspected outbreak	0.82 (0.79–0.85)	0.43 (0.37–0.49)	0.89 (0.87–0.91)	0.42 (0.37–0.47)
Census region				
South	Referent	Referent	Referent	Referent
Northeast	0.77 (0.74–0.81)	0.44 (0.37–0.52)	0.88 (0.85–0.90)	0.45 (0.38–0.53)
Midwest	1.07 (1.02–1.11)	1.02 (0.87–1.20)	1.00 (0.98–1.03)	1.03 (0.88–1.20)
West	0.99 (0.93–1.05)	0.95 (0.76–1.19)	0.97 (0.93–1.00)	0.94 (0.75–1.17)
Year				
Jan 2009–Jun 2009	1.34 (1.21–1.48)	2.49 (1.73–3.62)	1.20 (1.12–1.28)	2.50 (1.73–3.63)
Jul 2009–Jun 2010	1.37 (1.25–1.52)	2.59 (1.82–3.73)	1.22 (1.15–1.30)	2.55 (1.79–3.65)
Jul 2010–Jun 2011	1.34 (1.25–1.45)	2.64 (2.01–3.49)	1.20 (1.14–1.25)	2.58 (1.97–3.39)
Jul 2011–Jun 2012	1.24 (1.15–1.33)	2.12 (1.63–2.77)	1.14 (1.09–1.19)	2.16 (1.66–2.81)

Characteristics	RR of attack rates (95% CI)	OR of R_0>3.23 (95% CI)	Estimated log-linear change in R_e (95% CI)	OR of R_e>1.52 (95% CI)†
Jul 2012–Jun 2013	1.08 (1.01–1.15)	1.26 (0.99–1.59)	1.05 (1.01–1.09)	1.29 (1.02–1.63)
Jul 2013–Jun 2014	1.04 (0.97–1.11)	1.19 (0.93–1.52)	1.04 (1.00–1.07)	1.18 (0.93–1.51)
Jul 2014–Jun 2015	1.08 (1.01–1.15)	1.33 (1.05–1.68)	1.06 (1.02–1.10)	1.32 (1.04–1.66)
Jul 2015–Jun 2016	1.05 (0.98–1.12)	1.14 (0.91–1.44)	1.03 (1.00–1.07)	1.15 (0.91–1.44)
Jul 2016–Jun 2017	Referent	Referent	Referent	Referent
Jul 2017–Dec 2017	1.05 (0.96–1.14)	1.40 (1.03–1.90)	1.04 (0.99–1.09)	1.33 (0.98–1.80)

*Assuming the percent susceptible at the start of an outbreak is 47%.
†Logistic regression compares outbreaks with transmission in the third tertile (Re>1.52) to outbreaks in the first tertile (Re<1.17) and does not include Re values in second tertile. Linear and negative binomial regressions use full dataset.

Appendix Table 6. Estimated log-linear change in basic and effective reproduction numbers relative to the intercept from linear regression*

		Basic reproduction number		Effective reproduction number	
Characteristics	RR of attack rates (95% CI)	Estimated log-linear change (95% CI)	OR of R_0>3.23 (95% CI)†	Estimated log-linear change (95% CI)	OR of R_e>1.52 (95% CI)†
Intercept	0.28 (0.26–0.30)	3.39 (3.28–3.51)	2.01 (1.56–2.60)	1.63 (1.57–1.7)	2.00 (1.55–2.57)
Season					
Winter	Referent	Referent	Referent	Referent	Referent
Fall	0.99 (0.92–1.06)	1.00 (0.96–1.03)	0.92 (0.71–1.20)	1.00 (0.95–1.04)	0.90 (0.69–1.16)
Spring	0.96 (0.92–1.00)	0.97 (0.95–1.00)	0.87 (0.73–1.03)	0.97 (0.94–1.00)	0.87 (0.73–1.03)
Summer	0.83 (0.73–0.93)	0.90 (0.86–0.95)	0.55 (0.36-0.85)	0.90 (0.85–0.95)	0.54 (0.35–0.84)
Outbreak Status					
Confirmed outbreak	Referent	Referent	Referent	Referent	Referent
Suspected outbreak	0.81 (0.78–0.84)	0.89 (0.87–0.91)	0.39 (0.34-0.45)	0.89 (0.86–0.91)	0.39 (0.34–0.45)
Census Region					
South	Referent	Referent	Referent	Referent	Referent
Northeast	0.69 (0.66–0.73)	0.86 (0.84–0.88)	0.31 (0.25–0.37)	0.84 (0.82–0.86)	0.31 (0.26–0.38)
Midwest	1.00 (0.95–1.05)	0.98 (0.95–1.00)	0.83 (0.69–0.99)	0.99 (0.96–1.02)	0.81 (0.67–0.97)
West	0.96 (0.90–1.03)	0.96 (0.93–1.00)	0.89 (0.67–1.18)	0.96 (0.92–1.00)	0.89 (0.67–1.17)
Year					
Jan 2009–Jun 2009	1.47 (1.32–1.63)	1.20 (1.13–1.28)	3.39 (2.22–5.23)	1.26 (1.17–1.35)	3.41 (2.24–5.25)
Jul 2009–Jun 2010	1.46 (1.31–1.63)	1.21 (1.14–1.29)	3.04 (1.98–4.74)	1.28 (1.19–1.38)	2.92 (1.91–4.51)
Jul 2010–Jun 2011	1.43 (1.32–1.55)	1.20 (1.15–1.25)	3.27 (2.39–4.50)	1.25 (1.19–1.32)	3.13 (2.30–4.29)
Jul 2011–Jun 2012	1.28 (1.19–1.38)	1.14 (1.10–1.19)	2.39 (1.76–3.25)	1.17 (1.12–1.23)	2.35 (1.74–3.19)
Jul 2012–Jun 2013	1.10 (1.03–1.18)	1.05 (1.02–1.09)	1.35 (1.03–1.78)	1.07 (1.03–1.11)	1.35 (1.03–1.78)
Jul 2013–Jun 2014	1.08 (1.01–1.17)	1.04 (1.00–1.08)	1.29 (0.97–1.74)	1.05 (1.01–1.10)	1.28 (0.95–1.71)
Jul 2014–Jun 2015	1.16 (1.08–1.24)	1.07 (1.04–1.11)	1.59 (1.21–2.08)	1.09 (1.05–1.14)	1.56 (1.19–2.04)
Jul 2015–Jun 2016	1.08 (1.01–1.16)	1.02 (0.99, 1.06)	1.19 (0.9–1.58)	1.05 (1.00–1.09)	1.19 (0.90–1.57)
Jul 2016–Jun 2017	Referent	Referent	Referent	Referent	Referent
Jul 2017–Dec 2017	1.09 (0.99–1.20)	1.06 (1.01–1.12)	1.56 (1.08–2.27)	1.06 (1.00–1.13)	1.50 (1.04–2.16)

*Odds ratios of outbreaks with R_0>3.23 and R_e>1.52, final size adjusting for exposed population size among long-term care or assisted care facilities, assuming 47% susceptible at the start of the outbreak. R_0, basic reproduction number; R_e, estimated reproduction number.
†Logistic regression compares outbreaks with transmission in the third tertile (R_0>3.23, R_e>1.52) to outbreaks in the first tertile (R_0<2.48, R_e<1.17), and does not include R_e values in second tertile. Linear and negative binomial regressions use full dataset.

Appendix Table 7. Estimated log-linear change in basic reproductive number (R_0) from linear regression and odds ratios of outbreaks with high R_0 assuming 27% and 80% susceptible at the start of the outbreak*

	27% Susceptible		80% Susceptible	
Characteristics	Estimated log-linear change in R_0 (95% CI)	OR of R_0>8.05 (95% CI)†	Estimated log-linear change in R_0 (95% CI)	OR of R_0>1.54 (95% CI)†
Intercept	7.42 (7.18–7.68)	2.05 (1.66–2.54)	1.57 (1.54–1.60)	1.78 (1.43–2.22)
Major Setting				
Long-term care or assisted living facility	Referent	Referent	Referent	Referent
Child day care	0.92 (0.88–0.97)	0.64 (0.45–0.89)	1.02 (0.99–1.05)	1.11 (0.80–1.55)
Hospital or healthcare facility	0.85 (0.81–0.89)	0.46 (0.33–0.64)	0.99 (0.96–1.02)	0.66 (0.48–0.91)
Other	0.91 (0.87–0.95)	0.54 (0.40–0.72)	0.99 (0.96–1.02)	0.77 (0.58–1.01)
Private home or residence	0.53 (0.43–0.65)	0.02 (0.00–0.12)	1.17 (1.05–1.31)	5.17 (2.22–14.21)
Restaurant	0.69 (0.61–0.77)	0.17 (0.09–0.33)	1.08 (1.01–1.16)	1.78 (0.99–3.31)
School, college, or university	0.85 (0.82–0.88)	0.32 (0.25–0.39)	0.93 (0.91–0.94)	0.30 (0.24–0.38)
Season				
Winter	Referent	Referent	Referent	Referent
Fall	0.97 (0.94–1.01)	0.91 (0.74–1.12)	1.00 (0.98–1.01)	0.94 (0.77–1.16)
Spring	0.97 (0.94–0.99)	0.80 (0.69–0.93)	0.99 (0.98–1.00)	0.88 (0.76–1.02)
Summer	0.90 (0.86–0.95)	0.54 (0.39–0.75)	0.97 (0.94–1.00)	0.62 (0.45–0.85)
Outbreak Status				
Confirmed outbreak	Referent	Referent	Referent	Referent

Characteristics	27% Susceptible: Estimated log-linear change in R_0 (95% CI)	27% Susceptible: OR of R_0>8.05 (95% CI)†	80% Susceptible: Estimated log-linear change in R_0 (95% CI)	80% Susceptible: OR of R_0>1.54 (95% CI)†
Probable outbreak	0.87 (0.85–0.89)	0.45 (0.40–0.51)	0.95 (0.94–0.96)	0.42 (0.37–0.48)
Census Region				
South	Referent	Referent	Referent	Referent
Northeast	0.93 (0.91–0.96)	0.55 (0.47–0.65)	0.93 (0.92–0.95)	0.44 (0.37–0.52)
Midwest	0.99 (0.97–1.02)	0.93 (0.80–1.09)	1.01 (1.00–1.03)	1.03 (0.88–1.21)
West	1.03 (0.99–1.06)	1.10 (0.88–1.37)	0.98 (0.96–1.00)	0.95 (0.76–1.19)
Year				
Jan 2009–Jun 2009	1.12 (1.06–1.20)	2.05 (1.42–3.00)	1.10 (1.06–1.14)	2.62 (1.80–3.83)
Jul 2009–Jun 2010	1.11 (1.05–1.18)	1.86 (1.30–2.67)	1.12 (1.08–1.16)	2.64 (1.85–3.82)
Jul 2010–Jun 2011	1.11 (1.07–1.16)	1.95 (1.49–2.57)	1.09 (1.06–1.12)	2.59 (1.97–3.43)
Jul 2011–Jun 2012	1.13 (1.08–1.18)	2.09 (1.62–2.72)	1.06 (1.04–1.08)	2.15 (1.65–2.81)
Jul 2012–Jun 2013	1.02 (0.99–1.06)	1.13 (0.90–1.41)	1.02 (1.00–1.04)	1.33 (1.05–1.69)
Jul 2013–Jun 2014	1.01 (0.98–1.05)	1.12 (0.88–1.42)	1.02 (1.00–1.04)	1.20 (0.94–1.54)
Jul 2014–Jun 2015	1.03 (0.99–1.07)	1.26 (1.00–1.59)	1.03 (1.01–1.05)	1.37 (1.08–1.73)
Jul 2015–Jun 2016	1.00 (0.96–1.03)	1.01 (0.80–1.26)	1.03 (1.01–1.05)	1.12 (0.88–1.41)
Jul 2016–Jun 2017	Referent	Referent	Referent	Referent
Jul 2017–Dec 2017	1.04 (0.99–1.09)	1.22 (0.91–1.65)	1.01 (0.99–1.03)	1.31 (0.97–1.78)

*OR, odds ratio; R_0, basic reproduction number; R_e, estimated reproduction number.
†Logistic regression compares outbreaks with transmission in the third tertile (S = 27%(N): R_0>8.05; S = 80%(N): R_0>1.54) to outbreaks in the first tertile (S = 27%(N): R_0<4.96; S = 80%(N): R_0<1.36) and does not include R_0/R_e values in second tertile. Linear and negative binomial regressions use full dataset.

Appendix Table 8. Estimated log-linear change in R_e (95% CI) from linear regression and odds ratios of outbreaks with high R_e assuming 27% and 80% susceptible at the start of the outbreak

Characteristics	27% Susceptible: Estimated log linear change in R_e (95% CI)	27% Susceptible: OR of R_e>3.24 (95% CI)†	80% Susceptible: Estimated log-linear change in R_e (95% CI)	80% Susceptible: OR of R_e>1.23 (95% CI)†
Intercept	2.41 (2.31–2.52)	1.88 (1.51–2.33)	1.25 (1.23–1.28)	1.67 (1.34–2.07)
Major Setting				
Long-term care or assisted living facility	Referent	Referent	Referent	Referent
Child day care	0.95 (0.89–1.01)	0.79 (0.56–1.11)	1.01 (0.98–1.05)	1.05 (0.75–1.45)
Hospital or healthcare facility	0.85 (0.80–0.90)	0.41 (0.29–0.58)	0.99 (0.96–1.02)	0.63 (0.46–0.86)
Other	0.91 (0.86–0.97)	0.65 (0.48–0.86)	0.99 (0.96–1.02)	0.76 (0.58–1.00)
Private home or residence	0.98 (0.85–1.14)	0.86 (0.24–3.04)	1.24 (1.12–1.37)	8.47 (3.22–29.28)
Restaurant	0.90 (0.80–1.01)	0.35 (0.16–0.74)	1.08 (1.01–1.16)	1.53 (0.86–2.78)
School, college, or university	0.79 (0.75–0.82)	0.27 (0.21–0.34)	0.93 (0.91–0.94)	0.31 (0.25–0.39)
Season				
Winter	Referent	Referent	Referent	Referent
Fall	0.98 (0.94–1.02)	0.89 (0.72–1.10)	1.00 (0.98–1.01)	0.97 (0.79–1.19)
Spring	0.96 (0.93–0.99)	0.82 (0.71–0.95)	0.99 (0.97–1.00)	0.90 (0.77–1.04)
Summer	0.87 (0.82–0.93)	0.53 (0.37–0.73)	0.97 (0.94–1.00)	0.63 (0.46–0.86)
Outbreak Status				
Confirmed outbreak	Referent	Referent	Referent	Referent
Suspected outbreak	0.82 (0.80–0.84)	0.41 (0.36–0.47)	0.95 (0.94–0.96)	0.42 (0.37–0.48)
Census Region				
South	Referent	Referent	Referent	Referent
Northeast	0.86 (0.83–0.89)	0.47 (0.40–0.56)	0.93 (0.92–0.95)	0.45 (0.38–0.53)
Midwest	1.01 (0.98–1.04)	0.95 (0.81–1.12)	1.02 (1.00–1.03)	1.01 (0.86–1.18)
West	0.99 (0.95–1.04)	0.98 (0.78–1.23)	0.98 (0.96–1.00)	0.92 (0.74–1.15)
Year				
Jan 2009–Jun 2009	1.25 (1.16–1.35)	2.64 (1.83–3.85)	1.10 (1.06–1.15)	2.66 (1.84–3.88)
Jul 2009–Jun 2010	1.27 (1.18–1.36)	2.57 (1.80–3.69)	1.12 (1.08–1.16)	2.64 (1.86–3.78)
Jul 2010–Jun 2011	1.24 (1.17–1.31)	2.61 (1.99–3.43)	1.09 (1.07–1.12)	2.56 (1.95–3.36)
Jul 2011–Jun 2012	1.20 (1.14–1.27)	2.21 (1.70–2.87)	1.06 (1.04–1.09)	2.22 (1.71–2.89)
Jul 2012–Jun 2013	1.05 (1.01–1.10)	1.31 (1.03–1.65)	1.02 (1.01–1.04)	1.30 (1.03–1.65)
Jul 2013–Jun 2014	1.03 (0.99–1.08)	1.29 (1.01–1.65)	1.02 (1.00–1.04)	1.25 (0.98–1.59)
Jul 2014–Jun 2015	1.07 (1.02–1.12)	1.33 (1.06–1.69)	1.03 (1.01–1.05)	1.34 (1.06–1.69)
Jul 2015–Jun 2016	1.02 (0.98–1.07)	1.12 (0.88–1.41)	1.03 (1.01–1.05)	1.15 (0.91–1.45)
Jul 2016–Jun 2017	Referent	Referent	Referent	Referent
Jul 2017–Dec 2017	1.05 (0.99–1.12)	1.41 (1.04–1.91)	1.01 (0.99–1.03)	1.37 (1.01–1.86)

*OR, odds ratio; R_0, basic reproduction number; R_e, estimated reproduction number.
†Logistic regression compares outbreaks with transmission in the third tertile (S = 27%(N): R_e>3.24, S = 80%(N): R_e>1.23) to outbreaks in the first tertile (S = 27%(N): R_0<1.37, S = 80%(N): R_e<1.09), and does not include R_0/R_e values in second tertile. Linear and negative binomial regressions use full dataset.

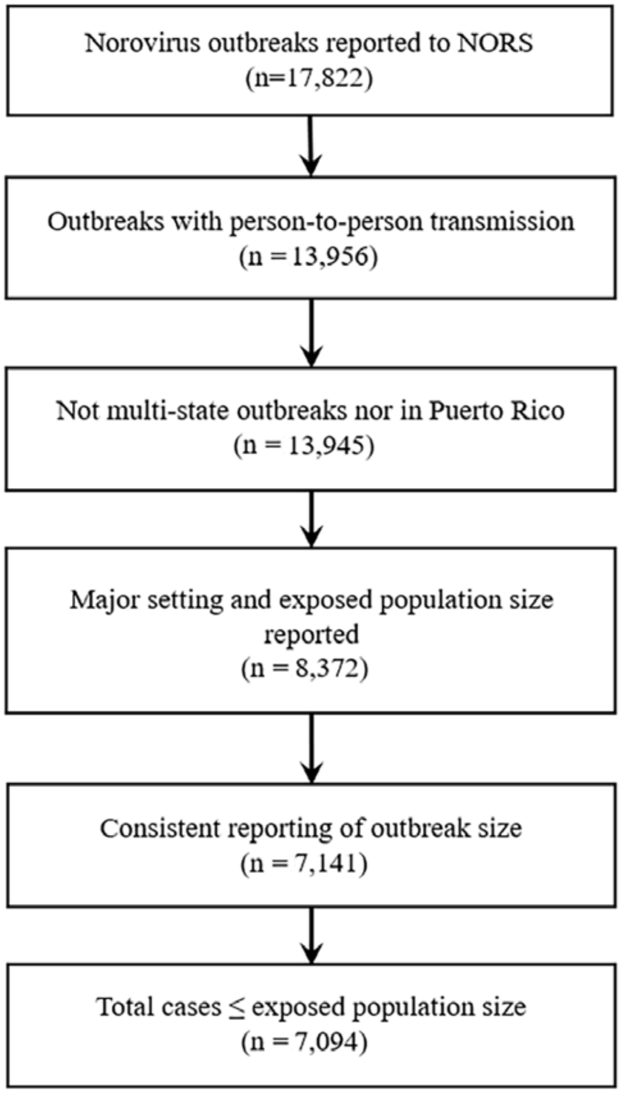

Appendix Figure 1. Flowchart of outbreaks included in analysis.

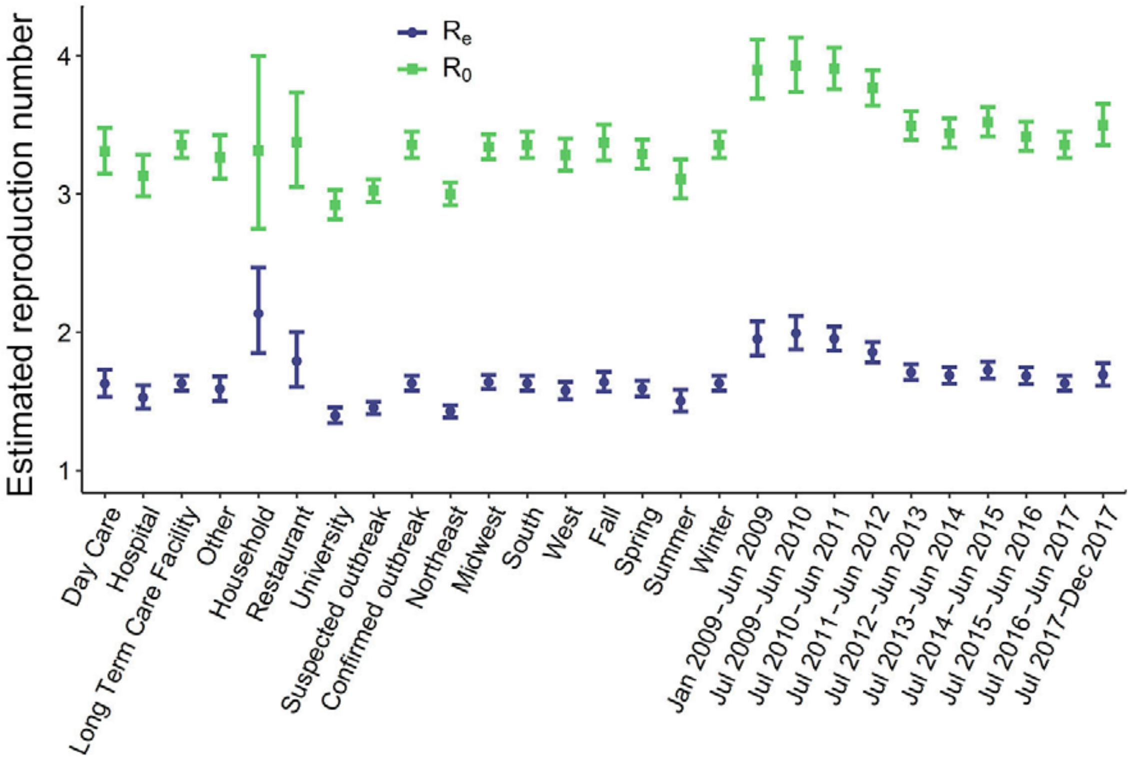

Appendix Figure 2. Reproduction rate, and estimated reproduction rate for norovirus in the United States assuming the initial proportion susceptible is 47%. Blue circles indicate estimated reproductive rate. Green circles indicate basic reproduction rate. R_0, basic reproduction rate; R_e, estimated reproduction rate.

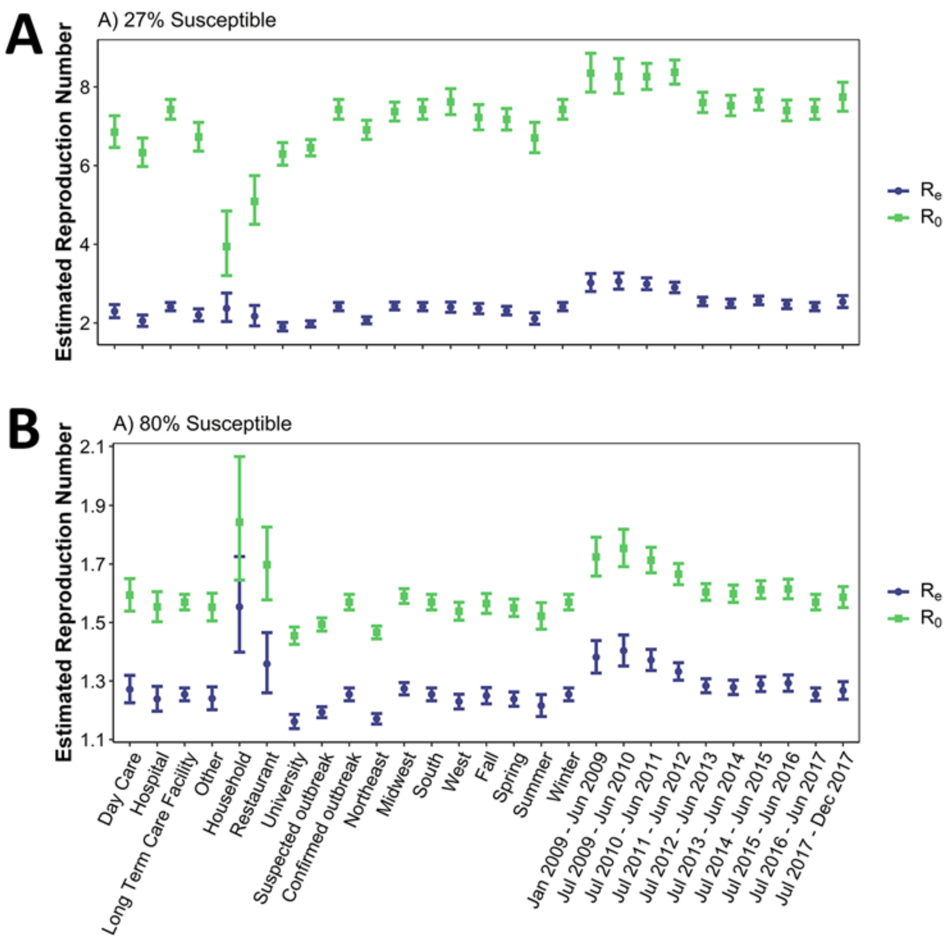

Appendix Figure 3. Estimated R_0 (green squares) and R_e (blue circles) assuming A) 27% susceptible and B) 80% susceptible at the start of the outbreak.

References

1. Becker NG. Analysis of infectious disease data. London: Chapman and Hall/CRC;1989.

2. Dolin R, Blacklow NR, DuPont H, Buscho RF, Wyatt RG. Kasel J a., et al. Biological properties of Norwalk agent of acute infectious nonbacterial gastroenteritis. Exp Biol Med. 1972 Jun;140:578–83. PubMed https://doi.org/10.3181/00379727-140-36508

3. Wyatt RG, Dolin R, Blacklow NR, DuPont HL, Buscho RF, Thornhill TS, et al. Comparison of three agents of acute infectious nonbacterial gastroenteritis by cross-challenge in volunteers. J Infect Dis. 1974;129:709–14. PubMed https://doi.org/10.1093/infdis/129.6.709

4. Parrino TA, Schreiber DS, Trier JS, Kapikian AZ, Blacklow NR. Clinical immunity in acute gastroenteritis caused by Norwalk agent. N Engl J Med. 1977;297:86–9. PubMed https://doi.org/10.1056/NEJM197707142970204

5. Treanor JJ, Madore HP, Dolin R. Development of an enzyme immunoassay for the Hawaii agent of viral gastroenteritis. J Virol Methods. 1988;22:207–14. PubMed https://doi.org/10.1016/0166-0934(88)90103-6

6. Johnson PC, Mathewson JJ, DuPont HL, Greenberg HB. Multiple-challenge study of host susceptibility to Norwalk gastroenteritis in US adults. J Infect Dis. 1990;161:18–21. PubMed https://doi.org/10.1093/infdis/161.1.18

7. Graham DY, Jiang X, Tanaka T, Opekun AR, Madore HP, Estes MK. Norwalk virus infection of volunteers: new insights based on improved assays. J Infect Dis. 1994;170:34–43. PubMed https://doi.org/10.1093/infdis/170.1.34

8. Lindesmith L, Moe C, Marionneau S, Ruvoen N, Jiang X, Lindblad L, et al. Human susceptibility and resistance to Norwalk virus infection. Nat Med. 2003;9:548–53. PubMed https://doi.org/10.1038/nm860

9. Lindesmith L, Moe C, Lependu J, Frelinger JA, Treanor J, Baric RS. Cellular and humoral immunity following Snow Mountain virus challenge. J Virol. 2005;79:2900–9. PubMed https://doi.org/10.1128/JVI.79.5.2900-2909.2005

10. Atmar RL, Opekun AR, Gilger MA, Estes MK, Crawford SE, Neill FH, et al. Norwalk virus shedding after experimental human infection. Emerg Infect Dis. 2008;14:1553–7. PubMed https://doi.org/10.3201/eid1410.080117

11. Leon JS, Kingsley DH, Montes JS, Richards GP, Lyon GM, Abdulhafid GM, et al. Randomized, double-blinded clinical trial for human norovirus inactivation in oysters by high hydrostatic pressure processing. Appl Environ Microbiol. 2011;77:5476–82. PubMed https://doi.org/10.1128/AEM.02801-10

12. Atmar RL, Bernstein DI, Harro CD, Al-Ibrahim MS, Chen WH, Ferreira J, et al. Norovirus vaccine against experimental human Norwalk Virus illness. N Engl J Med. 2011;365:2178–87. PubMed https://doi.org/10.1056/NEJMoa1101245

13. Seitz SR, Leon JS, Schwab KJ, Lyon GM, Dowd M, McDaniels M, et al. Norovirus infectivity in humans and persistence in water. Appl Environ Microbiol. 2011;77:6884–8. PubMed https://doi.org/10.1128/AEM.05806-11

14. Frenck R, Bernstein DI, Xia M, Huang P, Zhong W, Parker S, et al. Predicting susceptibility to norovirus GII.4 by use of a challenge model involving humans. J Infect Dis. 2012;206:1386–93. PubMed https://doi.org/10.1093/infdis/jis514

15. Bernstein DI, Atmar RL, Lyon GM, Treanor JJ, Chen WH, Jiang X, et al. Norovirus vaccine against experimental human GII.4 virus illness: a challenge study in healthy adults. J Infect Dis. 2015;211:870–8. PubMed https://doi.org/10.1093/infdis/jiu497
